# Distinct Origin of the Y and St Genome in *Elymus* Species: Evidence from the Analysis of a Large Sample of St Genome Species Using Two Nuclear Genes

**DOI:** 10.1371/journal.pone.0026853

**Published:** 2011-10-27

**Authors:** Chi Yan, Genlou Sun, Dongfa Sun

**Affiliations:** 1 College of Plant Science and Technology, Huazhong Agricultural University, Wuhan, Hubei, China; 2 Biology Department, Saint Mary's University, Halifax, Nova Scotia, Canada; Montreal Botanical Garden, Canada

## Abstract

**Background:**

Previous cytological and single copy nuclear genes data suggested the **St** and **Y** genome in the **StY**-genomic *Elymus* species originated from different donors: the **St** from a diploid species in *Pseudoroegneria* and the **Y** from an unknown diploid species, which are now extinct or undiscovered. However, ITS data suggested that the **Y** and **St** genome shared the same progenitor although rather few **St** genome species were studied. In a recent analysis of many samples of St genome species *Pseudoroegneria spicata* (Pursh) À. Löve suggested that one accession of *P. spicata* species was the most likely donor of the **Y** genome. The present study tested whether intraspecific variation during sampling could affect the outcome of analyses to determining the origin of **Y** genome in allotetraploid **StY** species. We also explored the evolutionary dynamics of these species.

**Methodology/Principal Findings:**

Two single copy nuclear genes, the second largest subunit of RNA polymerase II (RPB2) and the translation elongation factor G (EF-G) sequences from 58 accessions of *Pseudoroegneria* and *Elymus* species, together with those from *Hordeum* (**H**), *Agropyron* (**P**), *Australopyrum* (**W**), *Lophopyrum* (**E^e^**), *Thinopyrum* (**E^a^**), *Thinopyrum* (**E^b^**), and *Dasypyrum* (**V**) were analyzed using maximum parsimony, maximum likelihood and Bayesian methods. Sequence comparisons among all these genomes revealed that the **St** and **Y** genomes are relatively dissimilar. Extensive sequence variations have been detected not only between the sequences from **St** and **Y** genome, but also among the sequences from diploid **St** genome species. Phylogenetic analyses separated the **Y** sequences from the **St** sequences.

**Conclusions/Significance:**

Our results confirmed that **St** and **Y** genome in *Elymus* species have originated from different donors, and demonstrated that intraspecific variation does not affect the identification of genome origin in polyploids. Moreover, sequence data showed evidence to support the suggestion of the genome convergent evolution in allopolyploid **StY** genome species.

## Introduction

Hybridization and polyploidization have played an important role in the history of plant evolution, and contribute greatly to speciation [1]. Previous studies have reported that hybridization and chromosome doubling would create genetic shocks and the newly formed allopolyploids would need to undergo major intra- and inter-genomic changes. Many of these genome-wide alterations in allopolyploids could arise from rapid loss and recombination of low-copy DNA, retrotransposon activation, DNA methylation pattern changes and epigenetic gene silencing during or following polyploidization [2]–[4]. These rapid genomic changes may lead to genetic asymmetry evolution resulting in conformity and convergent effects caused by the inter-genome invasion of chromatin segments in either only one pair of chromosomes (chromosome-specific sequences) or in several chromosome pairs of one genome (genome-specific sequences) in the alloploids [5]–[10]. These changes may result in full fertility and stabilization of the hybrid condition and assist in establishing the phenotype in nature [11]. However, a clear and appropriate identification of phylogenetic relationships among taxa and genes, as well as genomic elements is needed.

The tribe Triticeae contains the world's most economically important grasses such as wheat, barley, and forage crops. The tribe combines a wide variety of biological mechanisms and genetic systems which make it an excellent group for research in evolution, and speciation [12]. *Elymus,* the largest genus in grass tribe Triticeae, includes approximately 150 species that are widely distributed all over the world. Moreover, *Elymus* is an exclusively allopolyploid genus but closely related to other genera in the Triticeae. Cytological analyses have identified five basic genomes (**St**, **H**, **Y**, **P**, and **W**) in this genus. It is believed that the **St** genome, found in all *Elymus* species, was donated by *Pseudoroegneria* (Nevski) Á. Löve, while the **H**, **P**, and **W** genomes are derived from *Hordeum* L., *Agropyron* Gaertn., and *Australopyrum* (Tzvelev) Á. Löve, respectively [13]–[16]. *Elymus* is an ideal genus for a genome duplication dynamic study because of the abundance of polyploid species and the close relationship to diploid taxa in the Triticeae,

Recently, one of the controversial debates about the *Elymus* genus is the origin of the **Y** genome. Previous studies have examined molecular evolution and phylogeny of some *Elymus* species [17]–[22], but it is still unclear where the **Y** genome originates from, although it is a common genome in Central and East Asia [14], [15], [23]. ITS sequence data suggest that **Y** and **St** may share the same progenitor genome [17], [18]. However, single copy of nuclear gene data of Mason-Gamer et al. [19], [20] and Sun et al. [21], [22] reject the idea that the **St** and **Y** genomes have the same origin. Instead, the results support Dewey's hypothesis that the **Y** genome had an independent origin from a **Y** diploid species that is now extinct or undiscovered. Okito et al. [24] employed a random amplified polymorphic DNA (RAPD) based sequence tagged site (STS) as a marker to investigate the origin of the **Y** genome using a relatively large number of *P. spicata* samples (**St** genome). The data showed that one accession of *P. spicata* (PI 232134) may be the donor of the **Y** genome and a prime candidate for the origin of the **Y** genome in *E. longearistatus* (**StY**). This conclusion is also consistent with the ITS data [18].

Phylogenetic analysis is routinely applied to test evolutionary questions, and tracing the origin of polyploidy based on interspecific data. This analysis generally assumes that intraspecifc variation is smaller than interspecific variation, and that within and between species, sample sizes are sufficiently large to capture variation at both levels [25]. Systematists deal with intraspecific variation in many different ways. For sequence analysis, they sample a single individual per species or treat each individual or haplotype as a separate terminal taxon [26]. This delineates the potential risk of bias. Whether the controversy on the origin of the **Y** genome in the **StY** *Elymus* species was caused by intraspecific variation during sampling or not still remains to be examined.

In the present study, we analyzed 16 accessions of **St** genome species *P. spicata*, 27 accessions of 14 other diploid species, and 15 allotetraploids representing the **StY** genome using two single copy nuclear genes: the translation elongation factor G (*EF-G*) and the second largest subunit of RNA polymerase II (*RPB2*). The objectives of this study are: (1) to confirm or reject whether **St** and **Y** share a common progenitor genome; (2) to explore whether intraspecific variation during the sampling would affect the result on the origin of **Y** genome in allotetraploid **StY** species; (3) to investigate *RPB2* and *EF-G* evolution in allopolyploid species and their potential donor species.

## Materials and Methods

### Plant materials and DNA extraction

Twenty-five accessions of 8 diploid species representing the **St** and other genomes were sequenced. The seeds were provided by USDA (United States Department of Agriculture). Germinated seeds were transplanted to a sand-peat mixture, and the plants maintained in a greenhouse. DNA was extracted from fresh young leaf tissues using the method of Junghans and Metzlaff [27]. *RPB2* and *EF-G* sequences for 15 polyploid Triticeae species representing the **StY** genome together with 17 accessions representing the **St**, **H**, **W**, **P**, **E** and **V** genomes, along with *Bromus sterilis* were obtained from published data [21], [22], and included in phylogenetic analyses. Plant material with accession numbers, genomic constitutions, geographical origins, and GenBank identification numbers are presented in Table S1. The species identity has confirmed for the accessions which we successfully obtained mature plants. The specimen were kept at Biology Department of Biology, Saint Mary's University.

### DNA amplification and sequencing

The single-copy nuclear genes *RPB2* and *EF-G* were amplified by polymerase chain reaction (PCR) using the primers P6F and P6FR [28], cMWG699T3-2 and cMWG699T7-2 [29], respectively. The amplification profile for the *RPB2* gene is as follows: an initial denaturation at 95°C for 4 min and 35–40 cycles of 95°C for 40 sec, 51°C for 30 sec, 72°C for 90 sec. The cycling ended with 72°C for 10 min. PCR profile for amplifying *EF-G* gene was based on Komatsuda et al. [29] except annealing temperature of 49°C. PCR products were purified using the QIAquickTM PCR purification kit (QIAGEN Inc.) according to manufacturer instructions.

PCR products were commercially sequenced by MACROGEN (Seoul, Korea). To enhance the sequence quality, both forward and reverse strands were sequenced independently. To avoid any error which would be induced by *Taq* DNA polymerase during PCR amplification, each sample was independently amplified twice and sequenced. *Taq* errors that cause substitutions are mainly random, and it is hence unlikely that the results of two sequences would share identical *Taq* errors to create a false synapomorphy.

### Data analysis

Automated sequence results were compared visually with chromatographs. Multiple sequence alignments were made using ClustalX with default parameters and additional manual edits to minimize gaps [30]. Phylogenetic analysis using the maximum-parsimony (MP) method was performed with the computer program PAUP* ver. 4 beta 10 [31]. All characters were specified as unweighted and unordered, and gaps were excluded in the analyses. Most-parsimonious trees were obtained by performing a heuristic search using the Tree Bisection-Reconnection (TBR) option with MulTrees selected, and ten replications of random addition sequences with the stepwise addition option. Multiple parsimonious trees were combined to form a strict consensus tree. Overall character congruence was estimated by the consistency index (CI), and the retention index (RI). In order to infer the robustness of clades, bootstrap values with 1000 replications [32] were calculated by performing a heuristic search using the TBR option with MulTrees on.

In addition to maximum parsimony analysis, maximum-likelihood (ML) and Bayesian analyses were performed. For ML analysis, 8 nested models of sequence evolution were tested for both the *RPB2* and *EF-G* data set using PhyML 3.0 [33]. For each data set, the general time-reversible (GTR) [34] substitution model led to a largest ML score compared to the other 7 substitution models: JC69 [35], K80 [36], F81 [37], F84 [38], HKY85 [39], TN93 [40] and custom (data not shown). As the result, the GTR model was used in the Bayesian analysis using MrBayes 3.1 [41]. Default uniform priors were used for all model parameters (six substitution rates, four base frequencies, proportion of invariable sites, and alpha value of gamma distribution). One cold and three incrementally heated Markov Chains Monte Carlo (mcmc) chains were run simultaneously, each for both the two sequences data with default heating value (0.2). In order to make the standard deviation of split frequencies fall below 0.01 so that the occurrence of convergence could be certain, 4,750,000 generations for *RPB2* data and 600,000 generations for *EF-G* were ran. Samples were taken every 1000 generations under the GTR model with gamma-distributed rate variation across sites and a proportion of invariable sites. For all analyses, the first 25% of samples from each run were discarded as burn in to ensure the stationarity of the chains. Bayesian posterior probability (PP) values were obtained from a majority rule consensus tree generated from the remaining sampled trees.

## Results

### Sequence variation

**Figure 1:**
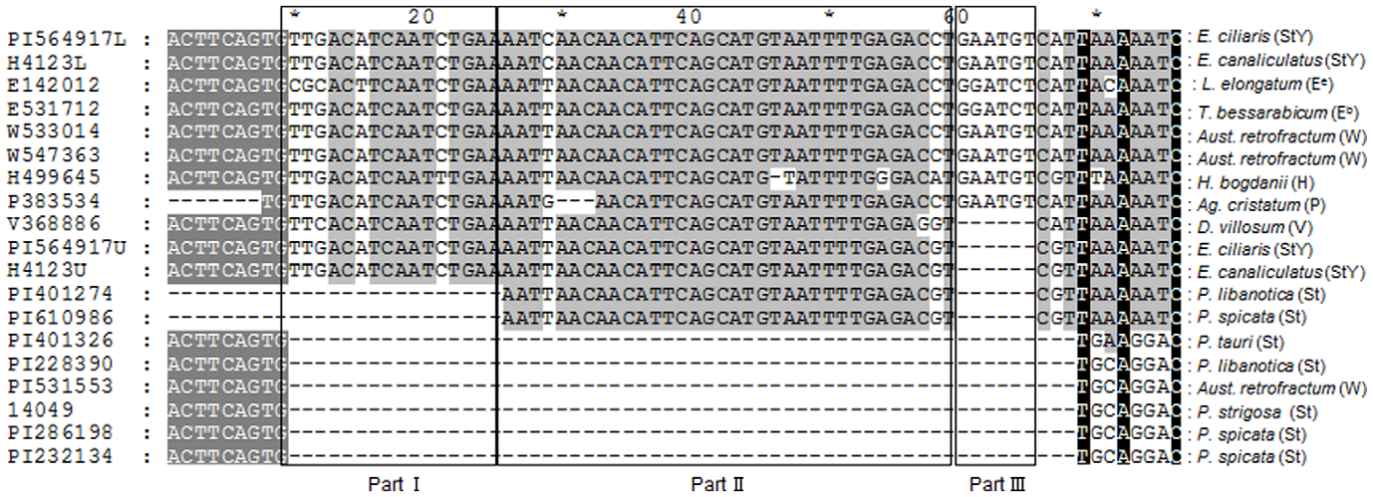
Partial alignment of amplified *RPB2* sequences from *Elymus* StY and their putative diploid donor species. Three uninterrupted indels showed in different boxes. (I): A 16 bp insertion were deleted in all diploid **St** genome sequences, two sequences from **W** genome (PI 533014 and PI 547363) and one sequence from **H** genome (PI 499645), compared to the **St** and **Y** sequences from allotetraploid **StY** species and all the sequences from **P**, **E^b^**, **E^e^** and **V** genome. (II): All sequences from diploid **St** species, except three *P. libanotica* accessions (PI 330687, PI 330688 and PI401274) and one *P. spicata* accession (PI 610986), have the 31 bp deletion compared to the sequences from **H**, **W** (except PI 531553), **P**, **E^b^**, **E^e^**, **V** and **StY** genome species. (III): A 6 bp insertion occurred in the **Y**, **H**, **W** (except PI 531553), **P**, and **E** genome sequences. None of the **St** genome contained the sequence (GAATGT) in this region.

The amplified patterns from each diploid species show a single band for both the *RPB2* and *EF-G* sequences with a size of approximately 800∼1000 bp, which corresponds well to previous findings [21], [22]. Not only have extensive sequence variations been detected in the present study between the sequences from the **St** and **Y** genomes, but also among the sequences from the diploid **St** genome species. Notably, sequence alignment shows a large insertion/deletion (indel) in the *RPB2* data (Fig. 1) which occurred at position 10. Some sequences were downloaded from Genbank and shorter than the others, so they were excluded for the comparison of this indel. In order to compare insertion/deletion among different species, we placed the data into three groups (boxes) (Fig. 1). The sequences in group I were deficient for all diploid **St** genome sequences and two sequences from the **W** genome (PI 533014 and PI 547363) together with one sequence from the **H** genome (PI 499645), compared to the **St** and **Y** sequences from allotetraploid **StY** species and all the sequences from **P**, **E^b^**, **E^e^** and **V** genomes. Group II included all sequences from diploid **St** species except the three *P. libanotica* accessions (PI 330687, PI 330688 and PI401274) and one *P. spicata* accession (PI 610986) had 31 bp deletions, while the sequences from **St** and **Y** genomes in tetraploid *Elymus* species, and the sequences from the **H**, **W** (except PI 531553), **P**, **E^b^**, **E^e^**, and **V** genome species did not have this deletion. Group III comprised samples with a 6 bp insertion that occurred in the **Y**, **H**, **W** (except PI 531553), **P**, and **E** genome sequences. None of the **St** genome contained the sequence GAATGT in this region.

### Phylogenetic analyses of *RPB2* sequences

**Figure 2:**
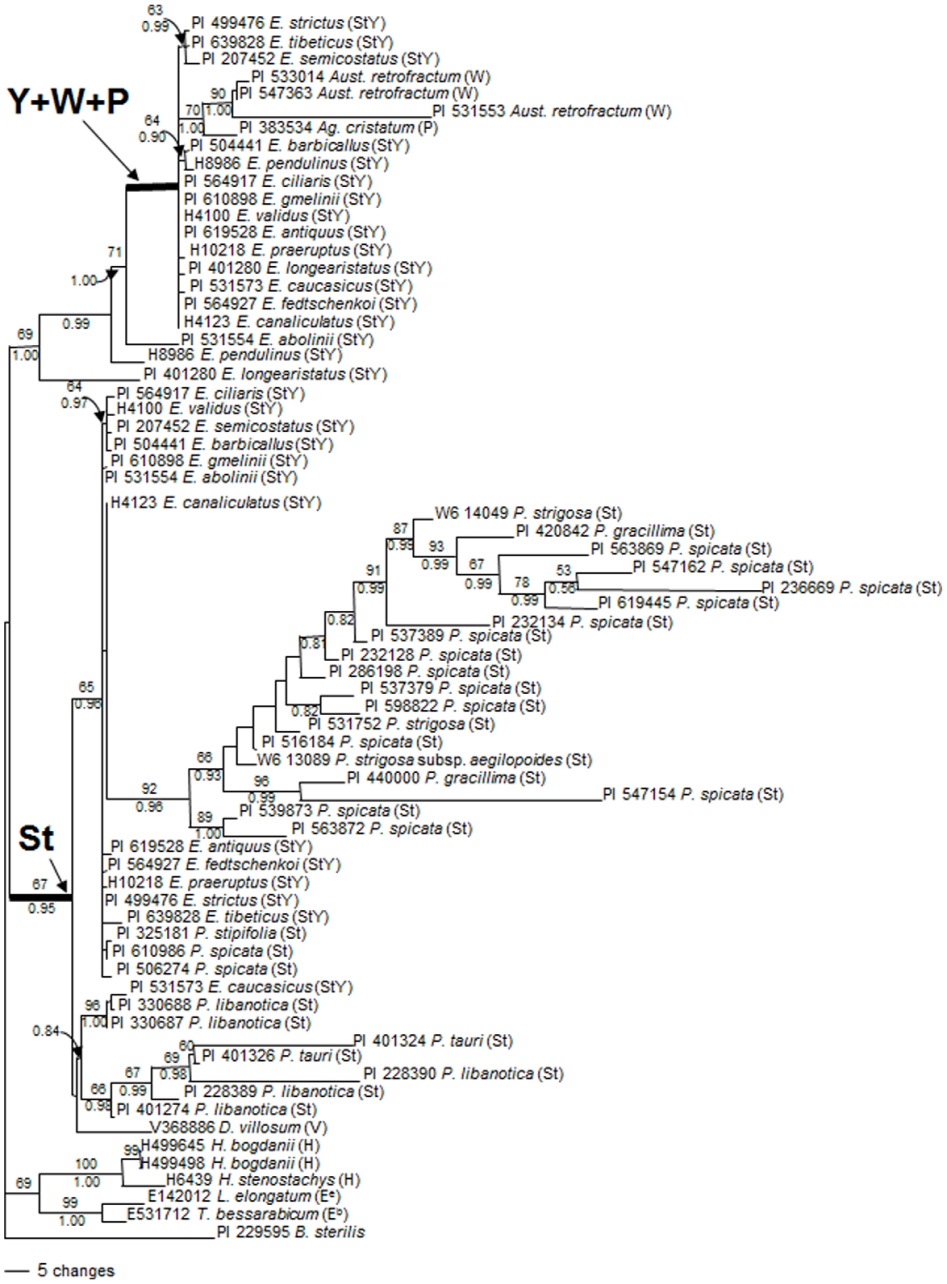
One of the 826 parsimonious trees derived from *RPB2* sequence data. The tree was conducted using heuristic search with TBR branch swapping. Numbers above and below branches are bootstrap values and Bayesian posterior probability (PP) values, respectively. Heavy internal branches are retained in the strict consensus tree. *Bromus sterilis* was used as an outgroup. Consistency index (CI)  = 0.650, retention index (RI)  = 0.836, rescale consistency index (RCI)  = 0.543.

Maximum parsimony analysis was conducted using *Bromus sterilis* as the outgroup. The parsimony analysis resulted in 826 equally most parsimonious trees (CI excluding uninformative characters  = 0.650; RI  = 0.836). The separated Bayesian analyses using GTR model resulted in identical trees with mean log-likelihood values −5318.82 and −5490.62 (data not shown). The tree topologies were almost identical in both ML and Bayesian likelihood trees and similar to those generated by MP, but only one of the most parsimonious trees with Bayesian PP and maximum parsimony bootstrap (1000 replicates) value is shown (Fig. 2).

Phylogenetic analyses separated the sequences into three clades. All diploid species from the **St** genome and one sequence of *D. villosum* representing the **V** genome were grouped together in 67% BS (PP  = 0.95). Included in the clade are the **St** genome sequences from tetraploid *Elymus* species (Fig. 2), while the **Y** copy sequences from tetraploid **StY** *Elymus* species formed a clade with the **W** and **P** genome species in 71% BS (PP  = 1.00). The two copies of sequences from each *Elymus* species except *E. pendulinus* (H8986) and *E. longearistatus* (PI 401280) were separated well into two different clades, while the sequence from *E. pendulinus* (H8986) and *E. longearistatus* (PI 401280) was related to the presumed **Y**+**W**+**P** genome clade. The only difference among the MP, ML and BI trees is the **Y** copy sequence of *E. semicostatus* (PI 207452) which separated from the subclade with two other **Y** copy from accessions PI 639828 and PI 499476 in the ML tree. These three sequences were grouped together and were fairly well supported by the BI and MP trees (63% BS, PP  = 0.99).

Within the **St** (*Pseudoroegneria + Elymus*) clade, all *P. spicata* accessions except PI 610986 and PI 506274 formed a well supported subclade (92% BS, PP  = 0.96), within which *P. strigosa* and *P. gracillima* were nested. The *P. spicata* sequences from accession PI 610986 and PI 506274, along with the **St** copy from **StY** tetraploid species were placed outside this subclade. The sequences from *P. tauri, P. libanotica* and *E. caucasicus* were grouped into a separate subclade.

### Phylogenetic analyses of the *EF-G* sequences

**Figure 3:**
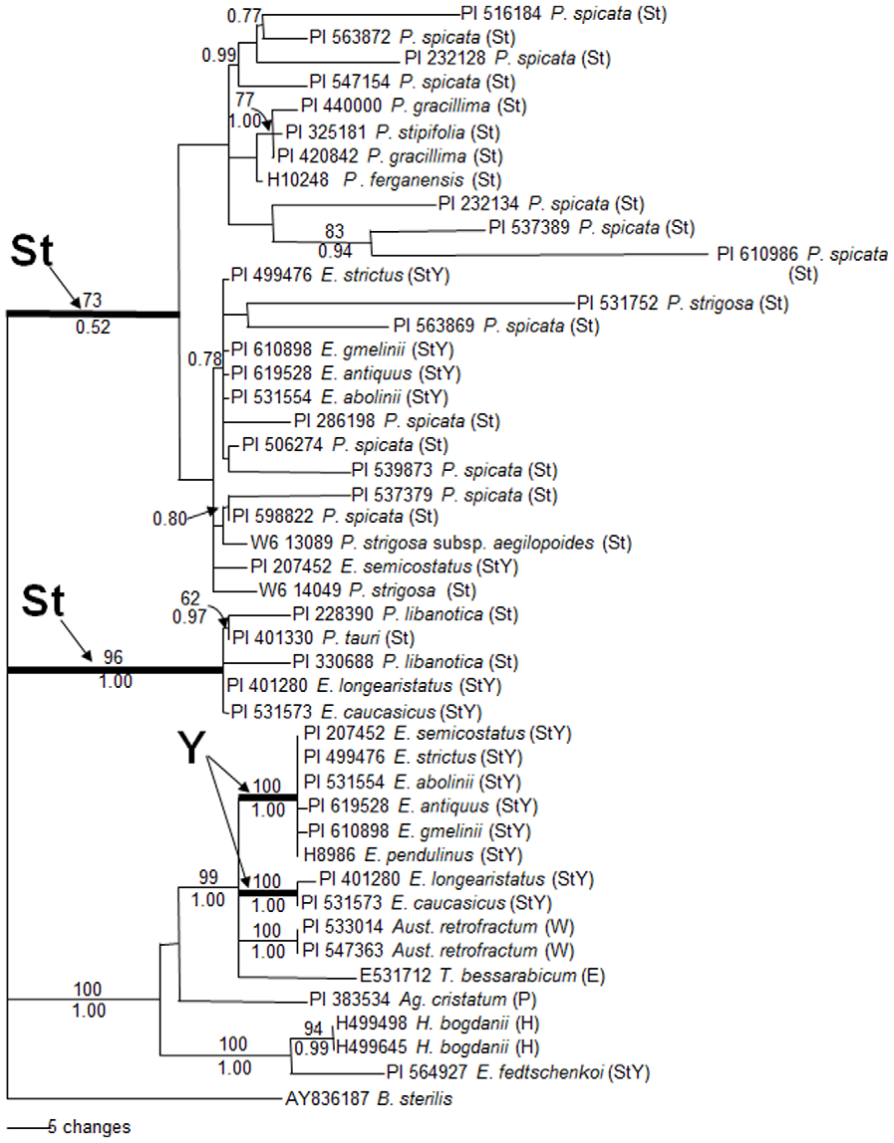
One of the 556 parsimonious trees derived from *EF-G* sequence data. The tree was conducted using heuristic search with TBR branch swapping. Numbers above and below branches are bootstrap values and Bayesian posterior probability (PP) values, respectively. Heavy internal branches are retained in the strict consensus tree. *Bromus sterilis* was used as an outgroup. Consistency index (CI)  = 0.863, retention index (RI)  = 0.906, rescale consistency index (RCI)  = 0.783.

The parsimony analysis resulted in 556 most parsimonious trees (CI excluding uninformative characters  = 0.863; RI  = 0.906). The Bayesian analyses using GTR model resulted in identical trees with mean log-likelihood values of −4132.80 and −4508.50 (data not shown). The tree topologies generated by ML, MP and Bayesian analyses were similar to each other, but only one of the most parsimonious trees with BS and PP values is shown in Figure 3.

In contrast to the *RPB2* sequences, Maximum Parsimony and Bayesian analyses based on *EF-G* sequences clearly separated the sequences from the **St** genome species into two distinct clades (Fig. 3). The first contained *P. tauri* and *P. libanotica*, as well as *E. longearistatus* and *E. caucasicus* with 96% BS support (PP  = 1.00) and the secont included *P. spicata*, *P. gracillima*, *P. stipifolia*, *P. ferganensis*, *P. strigosa* and *Elymus* species. However, the ML tree based on *EF-G* sequences still combined these two subclades into one large clade. The *P. spicata* accession PI 232134 that was suggested as the Y genome donor to *E. longearistatus* [24] was sister to the subclade (83% BS) of the other two *P. spicata* accessions (PI 537389 and PI 610986), and included in this *Pseudoroegneria + Elymus* clade.

All but one of the **Y** containing taxa with the **E** and **W** genomes formed a well supported clade (BS  = 99%, PP  = 1.00). The **Y** genome clade was further divided into two subclades. The first contained *E. semicostatus, E. strictus, E. abolinii, E. antiquus, E. pendulinus,* and *E. gmelinii* with 100% bootstrap support (PP  = 1.0). The second group comprised of *E. longearistatus* and *E. caucasicus* with 100% bootstrap support (PP  = 1.0). The **Y** copy sequence from *E. fedtschenkoi* (PI 564927) was not grouped into the **Y** genome clade and instead appeared as a sister to the **H** genome sequences.

## Discussion

### Possible origin of the Y genome and the relationships among the St and Y genome with other genomes in *Elymus* species

The phylogenetic analyses of *Elymus* and as many as 31 accessions from 8 **St** genome diploid species, in the present study, provide support for the distinct origin of the **Y** genome in polyploid **StY** species. Both *RPB2* and *EF-G* phylogenetic trees have well separated the **Y** genome from the **St** genome. These results are in accordance with the previous findings by Mason-Gamer et al. [19], [20] and Sun et al. [21], and support Dewey's hypothesis that there is a **Y** diploid species from which the **Y** genome originated. The data do not support the idea that the **St** and **Y** genomes have the same origin, which was based on ITS data by Liu et al. [18]. Recently, Okito et al. [24] suggested that one accession of *P. spicata* (PI 232134) might be the donor of the **Y** genome and a prime candidate for the origin of the **Y** genome to *E. longearistatus* (**StY**). In our study, the accession of *P. spicata* (PI 232134) was included but both *RPB2* and *EF-G* phylogenetic trees placed this accession in the **St** genome together with other *Pseudoroegneria* species. This indicates that there is not a close link between **St** genome in *P. spicata* and the **Y** genome in *E. longearistatus* or other **StY** genome species. Since the **Y** genome grouped with the **W** genome sequences in both the *RPB2* and *EF-G* trees, it implies that the **W** genome is closely related to the **Y** genome.

With respect to the relationships among different diploid species with the **St** genome, both the *RPB2* and *EF-G* data separated the *P. libanotica* + *P. tauri* group from other **St** genome species. The separation of *P. libanotica* from *P. spicata* was expected since previous AFLF analysis indicated a great difference between *P. libanotica* and *P. spicata* [42]. An interesting result in the *RPB2* tree was that the presumed **St** genome copy of *E. longearistatus* (**StY**) placed close to the **Y** genome clade with a 69% BS, rather than being grouped with other **St**-genome sequences from tetraploid **StY** species. Furthermore, in the *EF-G* tree, the presumed **St**-genome copy of *E. longearistatus* (**StY**) proved to be different from other larger **St**-gonome clade sequences and formed another well-supported clade (96% BS) with two *P. libanotica* individuals and *P. tauri*. This discrepancy within the sequence data between the **St** copy of *E. longearistatus* and the **St** genome clade sequences may be the reason why Okito et al. [24] suggested that one accession of *P. spicata* (PI 232134) could be the candidate donor of the **Y** genome to *E. longearistatus* (**StY**). However, since the sequences in the **St** genome of *E. longearistatus* are quite different from the other **St** genome sequences. In both the trees of *RPB2* and *EF-G* sequences, *P. libanotica* grouped with the **St** copy of *E. caucasicus* (PI 531573) and separated from other diploid **St** genome species. These data indicate that *P. libanotica* may be the donor of the **St** genome in allotetraploid *E. caucasicus* species.

### Dense sampling of intraspecies accession does not affect the identification of genome origin in polyploids

Intraspecific variation is abundant in all types of systematic characters which could cause bias in the phylogenetic analyses [43]. Systematists use different ways to deal with intraspecific variation [26]. There has been considerable debate as to which of the methods for directly analyzing polymorphic data is superior (e.g., [44]–[46]). One simulation study found that, overall, the most accurate methods were likelihood, the additive distance methods, and the frequency of parsimony method [26]. Okito et al. [24] used relatively large samples from *P. spicata* (**St** genome) to investigate the origin of the **Y** genome, and suggested that one accession of *P. spicata* (PI 232134) may be the donor of the **Y** genome, which conflicts the previous findings of Mason-Gamer et al. [19], [20], Sun et al. [21] and Sun and Komatsuda [22] who used a few samples from the **St** genomes species. In most previous phylogenetic studies, only one or two accessions have been used to represent entire species data, however, this neglects the change of intraspecific variation, and may result in a biased conclusion. This is the first time we used as many as 31 accessions from 8 **St** genome species in the phylogenetic analysis to evaluate if intraspecific variation could affect the phylogenetic result of the **Y** genome origin. MP, ML and Bayesian analyses reached the same conclusion that the **St** and **Y** genomes have distinct origins. Although fairly intraspecific variation has been detected in *P. spicata* [42] and other diploid species (data not shown), they do not influence the identification of the Y genome origin. It has been shown in previous studies that effective taxon sampling would be beneficial when analyzing the relationships across various levels of biological organization (e.g., genes, genomes, individuals, populations, species, or clades) due to poor taxon sampling leading to an increase in the apparent rate of variation which results in the overrepresentation of older nodes in the phylogenetic trees, and therefore, the bias caused by incomplete species sampling must be considered when using phylogenies to test hypotheses about species diversity (e.g., [47]–[51]). However, since the genome-wide recombination would have a much greater variation than the ones of intraspecies accessions, the sample size of each species would not affect our investigation of the inter-genome questions, such as genome origination, if the minimal requirement (two or more representative accessions per species) is reached.

### Genome evolution in allopolyploid species

The process of polyploidy occurs in cells and organisms when there are more than two paired (homologous) sets of chromosomes. During or after the process of allopolyploidization, rapid sequence elimination and restructuring of low-copy DNA, cytosine methylation, as well as the changes of transposable element activation and epigenetic gene silencing in allopolyploids shape the genomes in plants [2]–[4]. In this study, extensive nucleotide changes and genome-wide indels have been found between the sequences of the diploid **St** species and the allopolyploid **StY** species. The present study shows a 47 bp insertions in all the **St** copies of the *RPB2* sequences from allotetraploid **StY** species and all the diploid **St** species lack this insertion(Fig. 1 Part I and II) except the three *P. libanotica* accessions (PI 330687, PI 330688 and PI401274) and one *P. spicata* accession (PI 610986). One possible scenario is that the **St** genome in tetraploid **StY** species was donated by these four accessions. However, the geographical distribution of *P. spicata* does not overlap with **StY** *Elymus* species, and phylogenetic analyses did not provide convincing evidence of that the accession PI 610986 of *P. spicata* and *P. libanotica* are the **St** donor species to all allotetraploid **StY** species analyzed here (Figs. 2 and 3).

Another possible scenario is that the **St** genome in *Elymus* species acquired this part of the sequence by the inter-genome invasion of chromatin segments from the **Y** genome to the **St** genome and abundant genome-wide recombination following the fusion of **St** and **Y** gametes, before or after the process of polyploidization. It is also possible that the noted indels are homopasious, however, since the *RPB2* gene data from other genomic diploid species contain the same insertion as well, we could not rule out the possibility that this is due to the gene introgression between the **St** genome and other diploid species from the **W**, **H** or **E** genomes. Furthermore, this theory of gene introgression wouldn't work for the following reasons. The *RPB2* sequence data indicated that the tetraploid species *E. pendulinus* (H8986) and *E. longearistatus* (PI 401280) representing **StY** genome exhibited a high similarity between the two copies from **St** and **Y** genomes. As the result, the **St** copies of *E. pendulinus* (H8986) and *E. longearistatus* (PI 401280) are sister with the presumed **Y**-genome clade (69% BS) in the MP, ML and Bayesian trees. It could be that the **St** and **Y** genome have a common origin, however, the majority of 13 other tetraploid **StY** species sequences contain distinct **St** and **Y** genomes. Hence, the similarities between the *RPB2* sequences in the **St** and **Y** genome in *E. pendulinus* (H8986) and *E. longearistatus* (PI 401280) could not be explained by the same origin of the **St** and **Y** genomes. Also, only one copy of *EF-G* sequence from *E. pendulinus* (H8986) was found even though more than ten clones were screened. Assuming no bias in cloning or PCR amplification, this gives a 99.9% chance of obtaining at least one copy of each of the two ancestral allelic types for the allotetraploid [52]. Gene introgression between the **St** and **Y** genome species and other diploid species could not explain this. Genome-wide recombination between the **St** and **Y** genomes could result in the two genome sequences at this location being identical to the extent that we could not distinguish one from the other in this specific DNA fragment. Therefore, the explanation of genetic asymmetry evolution between the two parental genomes following polyploidization seems to be more likely than gene introgression.

Previous research has shown genome-wide recombination of allopolyploid between two constituent genomes in wheat species *T. turgidum* subsp. *dicoccoides* (Koern.) Thell., indicating that allopolyploid organisms can “select” the most efficient gene combination from one genome to control a set of related traits [11]. Comparative chromosomal studies using genetic mapping and fluorescence in situ hybridization (FISH) have demonstrated that inter-genome invasion of chromatin segments can occur from the **B** genome into the **A** genome (e.g., [53]). In the multiple independent synthetic lines of *Brassica napus* allotetraploids, genetic asymmetry evolution has been reported by Gaeta et al. [54], who found that convergent evolution of the two parental genomes could reflect extensive genomic combination. In contrast to the multiple copy genes, such as ITS, single-copy genes may more easily suffer from the loci loss due to random events after polyploidization [55]. Our finding using **StY** genome species support these ideas for the genome convergent evolution in allopolyploids.. Based on the two nuclear gene sequence data, we still could not determine the exact origin and the clear location of the **Y** genome in the tribe Triticeae Since sequence variability has been found in *E. pendulinus* and *E. longearistatus* more research is needed, to reveal the phylogenetic relationships and genome convergence in these species. Clearly, there are still a number of aspects which requires further study.

## Supporting Information

Table S1 Taxa from Bromus, Elymus, Hordeum, Pseudoroegneria, Lophopyrum, Thinopyrum, Agropyron, Australopyrum and Dasypyrum used in this study.
(DOC)
