# Supplementary material for: Distinct Origin of the Y and St Genome in Elymus Species: Evidence from the Analysis of a Large Sample of St Genome Species Using Two Nuclear Genes
Source: PLoS One. 2011 Oct 27;6(10):e26853. doi: 10.1371/journal.pone.0026853 (PMC3203181; doi:10.1371/journal.pone.0026853)
Supplement: Table S1 — Taxa from Bromus, Elymus, Hordeum, Pseudoroegneria, Lophopyrum, Thinopyrum, Agropyron, Australopyrum and Dasypyrum used in this study. (DOC) [file pone.0026853.s001.doc]

**Table S1.** Taxa from *Bromus, Elymus*, *Hordeum*, *Pseudoroegneria*, *Lophopyrum*, *Thinopyrum*, *Agropyron*, *Australopyrum* and *Dasypyrum* used in this study

| Species | Accession No. | Genome | RPB2 | EF-G |
| --- | --- | --- | --- | --- |
| *Ag. cristatum* (L.) Gaertn. | PI 383534 | P | EU187438 | GU982325 |
| *Aust. retrofractum* (Vickery) Á. Löve | PI 533014 | W | EU187482 | GU982345 |
|  | PI 547363 | W | EU187470 | GU982347 |
|  | PI 531553 | W | HQ231849 | - |
| *B. sterilis* L. | PI 229595 |  | HQ231839 | - |
|  | 55777 |  | - | AY836187 |
| *D. villosum* (L.) P. Candargy | PI 368886 | V | EU187471 | - |
| *H. bogdanii* Wilensky | PI 499498 | H | EF596768 | GU982334 |
|  | PI 499645 | H | EU18747 | GU982335 |
| *H. stenostachys* Godr. | H6439 | H | EU187473 | - |
| *P. ferganensis* (Nevski) Á. Löve | H10248 | St | - | GU982369 |
| *P. libanotica* (Hack.) D. R. Dewey | PI 330688 | St | EF596751 | HQ231866 |
|  | PI 330687 | St | EF596753 | - |
|  | PI 401274 | St | EF596752 | - |
|  | PI 228389 | St | HQ231837 | - |
|  | PI 228390 | St | HQ231838 | HQ231862 |
| *P. spicata* (Pursh) Á. Löve | PI 506274 | St | EF596746 | GU982338 |
|  | PI 610986 | St | EF596747 | GU982354 |
|  | PI 232128 | St | HQ231840 | HQ231863 |
|  | PI 232134 | St | HQ231841 | HQ231864 |
|  | PI 236669 | St | HQ231842 | - |
|  | PI 286198 | St | HQ231843 | HQ231865 |
|  | PI 516184 | St | HQ231848 | HQ231868 |
|  | PI 537379 | St | HQ231851 | HQ231870 |
|  | PI 537389 | St | HQ231852 | HQ231871 |
|  | PI 539873 | St | HQ231853 | HQ231872 |
|  | PI 547154 | St | HQ231854 | HQ231873 |
|  | PI 547162 | St | HQ231855 | - |
|  | PI 563869 | St | HQ231856 | HQ231874 |
|  | PI 563872 | St | HQ231857 | HQ231875 |
|  | PI 598822 | St | HQ231858 | HQ231876 |
|  | PI 619445 | St | HQ231859 | - |
| *P. stipifolia* (Czern. ex Nevski) Á. Löve | PI 325181 | St | EF596748 | GU982324 |
| *P. strigosa* (M. Bieb.) Á. Löve | PI 531752 | St | HQ231850 | HQ231869 |
|  | W6 14049 | St | HQ231836 | HQ231861 |
| *P. strigosa*(M. Bieb.) subsp. a*egilopoides*(Drobow) Á. Löve | W6 13089 | St | HQ231835 | HQ231860 |
| *P. gracillima* (Nevski) Á. Löve | PI 420842 | St | HQ231846 | GU982329 |
|  | PI 440000 | St | HQ231847 | HQ231867 |
| *P. tauri* (Boiss. & Balansa) Á. Löve | PI 401324 | StP | HQ231844 | - |
|  | PI 401326 | StP | HQ231845 | - |
|  | PI 401330 | StP | - | GU982328 |
| *L. elongatum* (Host) Á. Löve | PI 142012 | E^e^ | EU187439 | - |
| *T. bessarabicum* (Savul. & Rayss) Á. Löve | PI 531712 | E^b^ | EU187474 | GU982344 |
| *E. abolinii* (Drobow) Tzvelev | PI 531554 | StY | EU187443 | GU982339 |
|  |  |  | EU187444 | GU982340 |
| *E. ciliaris* (Trin.) Tzvelev | PI 564917 | StY | EF596749 | - |
|  |  |  | EU187483 | - |
| *E. semicostatus* (Nees ex Steud.) Melderis | PI 207452 | StY | EU187445 | GU982318 |
|  |  |  | EU187446 | GU982319 |
| *E. longearistatus* (Boiss.) Tzvelev | PI 401280 | StY | EU187447 | GU982326 |
|  |  |  | EU187448 | GU982327 |
| *E. canaliculatus* (Nevski) Tzvelev | H4123 | StY | EU187449 | - |
|  |  |  | EU187450 | - |
| *E. gmelinii* (Ledeb.) Tzvelev | PI 610898 | StY | EU187451 | GU982352 |
|  |  |  | EU187452 | GU982353 |
| *E. caucasicus* (Koch) Tzvelev | PI 531573 | StY | EU187453 | GU982342 |
|  |  |  | EU187454 | GU982343 |
| *E. pendulinus* (Nevski) Tzvelev | H8986 | StY | EU187455 | GU982367 |
|  |  |  | EU187456 | - |
| *E. praeruptus* Tzvelev | H10218 | StY | EU187459 | - |
|  |  |  | EU187460 | - |
| *E. strictus* (Keng) Á. Löve | PI 499476 | StY | EU187457 | GU982330 |
|  |  |  | EU187458 | GU982331 |
| *E. validus* (Meld.) B. Salomon | H4100 | StY | EU187461 | - |
|  |  |  | EU187462 | - |
| *E. antiquus* (Nevski) Tzvelev | PI 619528 | StY | EU187463 | GU982355 |
|  |  |  | EU187464 | GU982356 |
| *E. barbicallus* (Ohwi) S.L.Chen | PI 504441 | StY | EU187465 | - |
|  |  |  | EU187466 | - |
| *E. fedtschenkoi* Tzvelev | PI 564927 | StY | EU187467 | GU982349 |
|  |  |  | EU187468 | - |
| *E. tibeticus* (Meld.) G. Singh | PI 639828 | StY | EU187469 | - |
|  |  |  | EU187481 | - |
|  | | | | |
